# Analysis of postoperative atrial fibrillation and its associated factors in Morrow procedures with cardiopulmonary bypass

**DOI:** 10.1186/s12872-025-04514-0

**Published:** 2025-01-31

**Authors:** Zhibin Hu, Wenshuai Mao, Lijun Guo, Zhiwei Liu, Xujie Hu, Yong Cui

**Affiliations:** Heart Center, Department of Cardiovascular Surgery, Zhejiang Provincial People’s Hospital (Affiliated People’s Hospital, Hangzhou Medical College), No. 158 Shangtang Road, Hangzhou, Zhejiang 310014 China

**Keywords:** Atrial fibrillation, Cardiopulmonary bypass, Thoracoscopic, Minimally invasive, Systemic immune-inflammation index

## Abstract

**Background:**

The factors influencing the onset of new atrial fibrillation following the Morrow procedure due to cardiopulmonary bypass (CPB) are unclear. This study investigated the CPB-related factors associated with postoperative atrial fibrillation (POAF) in patients undergoing minimally invasive ventricular septal myectomy (Morrow procedure) to optimize CPB strategies, reduce the incidence of POAF, and enhance recovery.

**Methods:**

A retrospective clinical data analysis was conducted on 139 patients who underwent minimally invasive Morrow procedures from January to December 2023. The patients were divided into two groups based on whether they developed new-onset atrial fibrillation after surgery, and a comparative study was performed. Multivariate regression analysis were used to assess factors potentially influencing POAF during CPB.

**Results:**

Fifty (36%) patients developed POAF. Comparisons between the POAF group and the non-POAF group revealed significant differences in preoperative hypertension (38.0% vs. 14.6%, *p* = 0.002), ischaemic cardiomyopathy (40.0% vs. 20.2%, *p* = 0.012), history of heart failure (44.0% vs. 22.5%, *p* = 0.008), age (55.16 ± 14.11 vs. 46.28 ± 14.55, *p* = 0.001), the preoperative systemic immune-inflammation index (SII) (418.26 ± 243.97 vs. 330.24 ± 152.89, *p* = 0.019), the left atrial volume index (LAVI) (36.79 ± 12.08 vs. 32.24 ± 10.78, *p* = 0.024), CPB time (129.80 ± 39.58 vs. 116.96 ± 28.80, *p* = 0.027), CPB weaning time (25.68 ± 22.56 vs. 19.49 ± 6.78, *p* = 0.018), rate of re-CPB (14.0% vs. 3.4%, *p* = 0.020), rate of ultrafast-track cardiac anesthesia (UFTCA) (78.0% vs. 98.9%, *p* = 0.000), and ΔSII (2874.58 ± 2865.98 vs. 1981.85 ± 1519.89, *p* = 0.006) (*P* < 0.05). All patients were discharged, but the ICU (2.07 ± 2.91 vs. 1.38 ± 0.78, *p* = 0.046) and postoperative hospital stays (11.84 ± 7.50 vs. 9.13 ± 2.62, *p* = 0.002) were significantly prolonged. The results of the multivariate logistic regression analysis indicated that the occurrence of POAF was independently associated with age (OR = 1.047, 95% CI: 1.015–1.080), ΔSII(OR = 13.317, 95% CI: 3.103–57.154) and UFTCA(OR = 0.054, 95% CI: 0.006–0.493) (*p* < 0.05). Additionally, the increased value of SII was independently associated with CPB weaning time (t = 2.493, *p* = 0.014) and age(t=-2.270, *p* = 0.025).

**Conclusion:**

UFTCA is a protective factor against POAF. Age and ΔSII are risk factors for the occurrence of POAF after the Morrow procedure. CPB weaning time and Age are significant influencing factors of ΔSII. Implementing UFTCA and shortening the CPB weaning time are expected to lower the risk of POAF, shorten ICU and hospital stays, and enhance recovery.

**Clinical trial number:**

Not applicable.

## Background

Postoperative atrial fibrillation (POAF) is a common complication of cardiac surgery, with a significant variation in incidence according to surgery type [1]. The occurrence of POAF after cardiac surgery is markedly greater than that after noncardiac surgeries, ranging from 15 to 65%, and can reach as high as 30–65% following valve surgeries [2, 3]. These data, along with those of multiple studies, highlight the critical roles of surgical trauma, cardiopulmonary bypass (CPB), and inflammatory responses in the occurrence of POAF, emphasizing the necessity for an in-depth analysis of these risk factors [4, 5]. Hypertrophic obstructive cardiomyopathy (HOCM) is a condition characterized by abnormal thickening of the left ventricular myocardium, leading to left ventricular outflow tract obstruction. Ventricular septal myectomy (Morrow procedure) is a classic surgical method for treating HOCM that effectively improves blood flow and alleviates patient symptoms. However, the occurrence of POAF in HOCM patients post-surgery may increase the risk of serious consequences such as sudden death [6]. In light of this information, we conducted a study focusing on the occurrence of POAF after the minimally invasive Morrow procedure. This study particularly investigated CPB-related factors during surgery, explored their potential connections to the occurrence of POAF and identified key risk factors. Since CPB is a controllable technique, understanding its relationship with the development of POAF is crucial. The findings of this study are reported below.

## Materials and methods

### Definition of POAF

The Society of Thoracic Surgeons (STS) defines POAF as new atrial fibrillation lasting more than 1 h and/or requiring treatment [7].

### Clinical data

This study is a retrospective observational study. The subjects included 154 patients with HOCM treated in our department from January 2023 to December 2023. We excluded 10 patients with a history of atrial fibrillation, 2 patients who underwent surgery with hypothermic ventricle fibrillation, and 3 patients who were discharged automatically post-surgery, leaving 139 patients for the final analysis. All patients underwent a modified Morrow procedure through a thoracoscopic-assisted right axillary small incision via the aorta under general anaesthesia with peripheral cardiopulmonary bypass [8–10]. We meticulously collected preoperative data, including but not limited to age, sex, body surface area (BSA), medical history, comorbidities, and functional indicators of the heart and other vital organs. Intraoperative data included key parameters such as CPB time, aortic cross-clamp (ACC) time, CPB weaning time, volume of fluid input and output, ultrafiltration volume, and total blood transfusion. Postoperative data included electrocardiograms, ICU stay duration, hospital stay duration, pacemaker implantation status, and clinical indicators such as ultrafast track cardiac anaesthesia (UFTCA), cardiac ultrasound, and the immune inflammatory index. Patients were divided into POAF and non-POAF groups based on the occurrence of atrial fibrillation after surgery, followed by detailed comparative analysis. The primary objective of this study was to identify the main influencing factors for the occurrence of POAF, particularly its correlation with the CPB. The secondary objective was to summarize potential adverse clinical outcomes that POAF patients may encounter during hospitalization.

This study was approved by the Ethics Committee of our hospital (Approval No. Zhe Ren Yi Lun Shen 2024 Yan Di 244), and informed consent was waived. All the data were obtained from our hospital’s electronic medical records system.

### Statistical analysis

Statistical analysis was performed via SPSS version 22.0. Continuous variables are expressed as the means ± standard deviations (‾x ± s), with group comparisons conducted via t-tests or analysis of variance for normally distributed variables and nonparametric tests for nonnormally distributed variables. Categorical data are expressed as “numbers (percentages) or medians (quartiles)”, with group comparisons performed via Pearson’s χ² test or Fisher’s exact test. Univariate and multivariate logistic regression analyses were performed to determine the main influencing factors of POAF. A p-value of less than 0.05 was considered statistically significant.

## Results

### Echocardiography results of the minimally invasive morrow procedure

Transthoracic echocardiography revealed that the maximum interventricular septal thickness decreased from 20.28 ± 4.54 mm to 14.65 ± 2.73 mm (95% CI: 4.74–6.51, *p* < 0.001), and the left ventricular outflow tract (LVOT) pressure gradient decreased from 86.08 ± 41.68 mmHg to 9.58 ± 5.54 mmHg (95% CI: 69.45–83.55, *p* < 0.001).

### Overall incidence of POAF

Among the 139 HOCM patients who underwent the minimally invasive Morrow procedure, 50 developed POAF, accounting for 36% of the total.

## Summary of clinical data between POAF patients and Non-POAF patients

### Preoperative data

There were significant differences between the POAF and non-POAF groups in terms of age, preoperative hypertension, ischaemic cardiomyopathy, history of heart failure, preoperative systemic immune-inflammation index (SII), and left atrial volume index (LAVI) (*P* < 0.05). No significant differences were found for the other indicators (*p* > 0.05) (Table 1).

**Table 1:** Comparison of Preoperative Data between the POAF and Non-POAF groups

	POAF(*n* = 50)	N-POAF(*n* = 89)	*P* value
Age(years)	55.16 ± 14.12	46.28 ± 14.55	0.001
Male[n(%)]	20(40.0%)	47(52.8%)	0.147
BSA(m^2^)	1.82 ± 0.20	1.85 ± 0.18	0.374
Hypertension[n(%)]	19(38.0%)	13(14.6%)	0.002
Diabetes[n(%)]	2(4.0%)	7(7.9%)	0.374
Ischaemic cardiomyopathy[n(%)]	20(40.0%)	18(20.2%)	0.012
Lung disease[n(%)]	10(20.0%)	15(16.9%)	0.643
Smoking[n(%)]	15(30.0%)	20(22.5%)	0.326
History of heart failure[n(%)]	22(44.0%)	20(22.5%)	0.008
EF1(%)	68.76 ± 5.12	70.25 ± 4.17	0.065
cTnI(µg/L)	0.154 ± 0.306	0.151 ± 0.358	0.664
BNP(pg/ml)	556.97 ± 653.08	564.04 ± 625.40	0.779
HB(g/L)	137.98 ± 16.74	137.79 ± 18.80	0.952
SII(×10^9^/L)	418.26 ± 243.97	330.24 ± 152.89	0.019
LAVI(ml/m^2^)	36.79 ± 12.08	32.24 ± 10.78	0.024

EF1: Preoperative Left Ventricular Ejection Fraction; cTnI: Cardiac Troponin I; BNP: B-type Natriuretic Peptide; HB: Haemoglobin; SII: Systemic Immune-Inflammation Index; LAVI: Left Atrial Volume Index

### Perioperative data

Comparing the POAF group with the non-POAF group, there were significant differences in CPB time, CPB weaning time, rate of re-CPB, and rate of UFTCA (*P* < 0.05). No significant differences were observed in the other perioperative indicators (*p* > 0.05) (Table 2).

**Table 2:** Comparison of perioperative data between the POAF and non-POAF groups

	POAF(*n* = 50)	N-POAF(*n* = 89)	*P* value
CPB time (min)	129.80 ± 39.58	116.96 ± 28.80	0.027
ACC time (min)	81.56 ± 22.57	76.93 ± 23.51	0.164
CPB weaning time (min)	25.68 ± 22.56	19.49 ± 6.78	0.018
re-CPB[n(%)]	7(14.0%)	3(3.4%)	0.020
Ultrafiltration volume (ml)	1730.00 ± 745.39	1824.72 ± 736.44	0.470
Urine volume (ml)	401.00 ± 250.36	394.72 ± 320.97	0.770
NET volume (ml)	103.60 ± 741.81	-51.69 ± 744.50	0.239
blood transfusion [n(%)]	21(42.0%)	25(28.1%)	0.094
UFTCA [n(%)]	39(78.0%)	88(98.9%)	0.000

CPB: Cardiopulmonary Bypass; ACC: Aortic Cross-Clamping. UFTCA: ultrafast-track cardiac anaesthesia

### Postoperative adverse clinical outcomes

Compared with that in the non-POAF group, the ΔSII (increased value of SII post-surgery) in the POAF group was significantly greater (*P* < 0.05); no other postoperative indicators were significantly different (*P* > 0.05). The ICU stay and postoperative hospital stay in the POAF group were significantly longer than those in the non-POAF group (*P* < 0.05) (Table 3).

**Table 3:** Comparison of postoperative indicators and adverse clinical outcomes between the POAF and non-POAF groups

	POAF(*n* = 50)	N-POAF(*n* = 89)	*P* value
EF2(%)	60.94 ± 6.91	61.70 ± 5.49	0.479
cTnI(µg/L)	15.03 ± 15.56	17.96 ± 18.57	0.629
BNP(pg/ml)	762.33 ± 724.43	788.11 ± 842.13	0.452
HB(g/L)	118.04 ± 16.48	117.76 ± 15.45	0.922
ΔSII(×10^9^/L)	2874.58 ± 2865.98	1981.85 ± 1519.89	0.006
drainage volume	306.80 ± 386.70	261.97 ± 210.51	0.754
ICU stay(d)	2.07 ± 2.91	1.38 ± 0.78	0.046
postoperative hospital stay(d)	11.84 ± 7.50	9.13 ± 2.62	0.002

EF2: Postoperative Left Ventricular Ejection Fraction; cTnI: Cardiac Troponin I; BNP: B-type Natriuretic Peptide; HB: Haemoglobin; ΔSII: Increased value of Systemic Immune-Inflammation Index post-surgery; LAVI: Left Atrial Volume Index; ICU: Intensive Care Unit

### Multivariate logistic regression analysis

After univariate analysis, we performed a multivariate binary regression analysis using the conditional forward method on the indicators significantly related to the occurrence of POAF. The results indicated that the occurrence of POAF was associated with age (OR = 1.047, 95% CI: 1.015–1.080), ΔSII (OR = 13.317, 95% CI: 3.103–57.154), and UFTCA (OR = 0.054, 95% CI: 0.006–0.493) (*p* < 0.05). UFTCA was a protective factor against POAF, whereas age and ΔSII were risk factors for POAF (Table 4).

**Table 4:** Multivariate logistic regression analysis of POAF

	B	*P* value	OR	95% CI
Age	0.046	0.004	1.047	1.015–1.080
ΔSII^#^	2.589	0.000	13.317	3.103–57.154
UFTCA	-2.911	0.010	0.054	0.006–0.493

UFTCA: ultrafast-track cardiac anaesthesia. ΔSII: Increased value of Systemic Immune-Inflammation Index post-surgery. #: log-normalized values

In addition, we analyzed the factors that may be associated with ΔSII. The results of the univariate and multivariate linear logistic regression analyses indicated that the CPB weaning time (t = 2.493, *p* = 0.014) and age(t=-2.270, *p* = 0.025) were independently associated with increased value of SII (Table 5).

**Table 5:** Multivariate linear logistic regression analysis of ΔSII

	β	t	*P* value
Age	-0.189	-2.270	0.025
CPB weaning time	0.208	2.493	0.014

CPB: Cardiopulmonary Bypass; UFTCA: ultrafast-track cardiac anaesthesia

## Discussion

Despite advancements in surgical concepts, perioperative care, and preventive pharmacotherapy, the incidence of POAF following cardiac surgery has not decreased significantly over the past few decades [11]. The incidence of POAF is lowest after isolated coronary artery bypass grafting (CABG), higher after isolated valve surgery, and highest after combined valve/CABG surgeries, indicating that the type of surgery and the use of CPB play crucial roles in the development of POAF [12, 13]. HOCM is a structural heart disease distinct from coronary artery disease and valvular disease and is characterized by abnormal thickening of the ventricular myocardium, leading to left ventricular outflow tract obstruction. The classical surgical treatment is septal myectomy, where a portion of the hypertrophied septal myocardium is resected to relieve left ventricular outflow obstruction. HOCM associated with arrhythmias is a common cause of sudden cardiac death; however, reports on the relationships among the Morrow procedure, CPB, and the development of arrhythmias in the treatment of HOCM are limited.

This study focused on patients with HOCM and conducted an in-depth analysis of the CPB-related factors associated with the occurrence of POAF following the Morrow procedure. These findings have important clinical implications for optimizing CPB strategies and reducing the incidence of POAF. This study identified several factors had significant differences between the POAF and non-POAF groups, including age, preoperative hypertension, ischaemic cardiomyopathy, history of heart failure, the SII, LAVI, CPB time, CPB weaning time, re-CPB, and UFTCA. And Age, ΔSII, UFTCA were independently associated with the occurrence of POAF in multivariate binary regression analysis. Notably, UFTCA was confirmed as a protective factor against POAF. Furthermore, the results of the multivariate linear logistic regression analyses indicated that ΔSII were independently associated with the CPB weaning time and age. These findings provide clinicians with targeted interventions to reduce the incidence of POAF, potentially shortening patients’ ICU and hospital stays and accelerating recovery.

The results of this study align with key findings in the literature, which identify older age, hypertension, and a history of heart failure as high-risk factors for POAF [5]. Interestingly, the study also revealed that the UFTCA may be a protective factor against POAF. UFTCA is an advanced anaesthesia management strategy used in cardiac surgery aimed at reducing surgical stress and trauma, facilitating early extubation, and promoting rapid recovery. Several studies have confirmed the benefits of UFTCA combined with minimally invasive techniques in cardiac surgery, including reduced hospital stays, lower complication rates, and improved patient satisfaction [9, 14–16]. However, there have been no previous reports on the impact of UFTCA on the incidence of POAF after cardiac surgery or its underlying mechanisms, making this study the first to provide evidence of this association.

SII is an emerging biomarker that provides an integrated measure of inflammation by incorporating neutrophil, lymphocyte, and platelet counts. In this study, the ΔSII was significantly correlated with the occurrence of POAF. The increase in the postoperative SII is closely associated with the occurrence of POAF, which aligns with the role of inflammation and oxidative stress in the pathogenesis of POAF [17, 18]. The increased SII may reflect the systemic inflammatory response induced by factors such as CPB, surgical trauma and more. Previous studies have addressed this issue. The prolonged CPB weaning time may be linked to exacerbated inflammatory responses. Our findings confirm this hypothesis, indicating that the duration of CPB weaning was associated with ΔSII. In cardiac surgery, the CPB weaning time, defined as the period from aortic unclamping to the end of CPB, is a critical phase during which the myocardium may suffer additional ischemia-reperfusion injury. The ischemia-reperfusion injury to cardiac tissue can lead to the release of inflammatory mediators. An extended weaning time may increase the accumulation of these inflammatory mediators, thereby increasing the SII. Furthermore, the prolonged CPB weaning time may also reflect the complexity of the surgical process, which can further exacerbate systemic inflammatory responses. Conversely, reducing the CPB weaning time and improving the quality of CPB weaning-off are effective measures to reduce ΔSII.

### Limitations of the study

Despite providing valuable insights, this study has several limitations. First, as a retrospective study, it may be subject to selection and information bias. Second, although our hospital is one of the major centers for HOCM treatment in China, the annual number of Morrow procedures remains relatively low, limiting the sample size available for research. Finally, this study did not explore the detailed mechanisms by which UFTCA acts as a protective factor, warranting further investigation in future studies.

### Future research directions

On the basis of the findings and limitations of this study, future research could focus on the following areas: (1) Prospective multicentre studies: By employing a prospective design and multicentre collaboration, future studies can increase the sample size and improve the generalizability and reliability of the results. (2) Mechanistic Research: Studies can investigate the biological mechanisms through which UFTCA reduces the incidence of POAF, including its potential effects on the inflammatory response and immune regulation. (3) Multifactorial intervention studies: Comprehensive intervention studies can be conducted that target identified risk factors and assess the impact of interventions on POAF incidence. These could include exploring strategies to reduce the CPB weaning time, as well as its effects on the SII and patient outcomes.

## Conclusion

This study analysed the factors related to POAF following minimally invasive Morrow procedure and suggested potential interventions for reducing the incidence of POAF, such as actively implementing UFTCA, shortening the weaning time of CPB. Despite its limitations, this study underscores the importance of optimizing perioperative management and provides directions for future research. With further studies that deepen the understanding of POAF pathogenesis, we hope to more effectively prevent POAF and improve the clinical outcomes of cardiac surgery patients.

## Data Availability

The datasets used and/or analysed during the current study are available from the corresponding author upon reasonable request.
